# High expression of wee1 is associated with malignancy in vulvar squamous cell carcinoma patients

**DOI:** 10.1186/1471-2407-13-288

**Published:** 2013-06-14

**Authors:** Gry Irene Magnussen, Ellen Hellesylt, Jahn M Nesland, Claes G Trope, Vivi Ann Flørenes, Ruth Holm

**Affiliations:** 1 Department of Pathology, The Norwegian Radium Hospital, Oslo University Hospital and University of Oslo, Oslo, Montebello 0310, Norway; 2 Department of Obstetrics and Gynecology, The Norwegian Radium Hospital, Oslo University Hospital and University of Oslo, Oslo, Norway

**Keywords:** Vulvar squamous cell carcinoma, Wee1, Treatment, Targeted therapy, Biomarkers

## Abstract

**Background:**

Vulvar squamous cell carcinoma is a cancer form with increasing incidence rate and few treatment options. Wee1 is a central regulator of the G2/M DNA-damage checkpoint, and has in previous studies been described as a prognostic biomarker and a potential target for therapy in other cancer forms.

**Methods:**

In the present study we analyzed the expression of Wee1 in a panel of 297 vulvar tumors by immunohistochemistry. Furthermore, siRNA transfections were carried out in two vulvar cancer cell lines (SW-954 and CAL-39) in order to study the effect on cell cycle distribution (flow cytometry) and proteins (western blot) involved in DNA damage response and apoptosis.

**Results:**

Wee1 kinase is increased in vulvar squamous cell carcinomas, as compared to expression in normal epithelium, and a high Wee1 expression is associated with markers of malignancy, such as lymph node metastasis and poor differentiation. Our in vitro results showed that siRNA mediated Wee1 silencing only led to a modest reduction in viability, when examined in vulvar cancer cell lines. Nonetheless, a marked increase in DNA damages, as assessed by augmented levels of γ-H2AX, was observed in both cell lines in the absence of Wee1.

**Conclusions:**

Our results suggest that Wee1 may be involved in the progression of vulvar carcinomas. Based on our in vitro results, Wee1 is unlikely to function as a target for mono-treatment of these patients.

## Background

Vulvar cancer is a relatively rare malignancy and comprises 3-5% of all female genital cancer, however as a consequence of an aging population the incidence rate has risen steadily with 20% over the past 40 years [1]. A total of 4340 new vulvar cancer cases and 940 deaths from this disease were estimated in the United States in 2011 [2]. The 5-year survival is 98% (stage I), 85% (stage II), 74% (stage III) and 31% (stage IV) [3]. The incidence of vulvar cancer has been linked to advancing age, but also appears in younger women [4]. Radical vulvectomy with bilateral inguinofemoral lymphadenectomy has been the standard treatment for most patients, but this carries significant side effects/burden of morbidity [5]. Therefore, the search for treatment alternatives with less radical surgery is ongoing. Thus, the identification of new biomarkers could be important for development of better treatment strategies and may improve the prediction of clinical outcome.

The Wee1 kinase is a central regulator of the G2/M cell cycle checkpoint. In cases of DNA damage Wee1 adds an inhibitory phosphorylation on the Tyr15 residue of CDK1, by so postponing progression to mitosis and giving the cell time to either correct the damage or undergo apoptosis [6]. Furthermore, recent studies have indicated a role of Wee1 in safeguarding the genome during S- phase, as inhibition of the kinase has led to replication stress and subsequent DNA damage [7,8]. Whereas the G1/S checkpoint is deregulated in the vast majority of human cancers, the G2/M checkpoint genes are rarely, if ever, mutated [6]. Inhibiting proteins involved in the G2/M checkpoint, such as Wee1, may therefore selectively target cancer cells whilst sparing normal cells with a functional G1/S checkpoint. Elevated levels of Wee1 have been reported in human glioblastoma, osteosarcoma, breast cancer and melanoma [9-12], whilst down-regulation, on the other hand, has been observed in non-small-cell lung cancer [13]. To our knowledge, Wee1 in vulvar tumors has not previously been reported. In the present study our aim was to determine Wee1 expression in vulvar cancer, if it had an association with known clinicopatological variables and biomarkers, and finally if in vitro targeting of the kinase may be beneficial as mono-therapy.

## Methods

### Patient materials

A total of 297 patients were diagnosed with vulvar squamous cell carcinoma between 1977 and 2006 at The Norwegian Radium Hospital. The median patient age at diagnosis was 74 (range 35–96) years. Prior to surgery, three patients received radiotherapy/chemotherapy whereas another six received radiotherapy. Radical surgery (total vulvectomy and a bilateral inguinal lymphadenectomy) was performed in 192 (65%) of these cases and the remaining 105 (35%) patients received non-radical surgery. Postoperative irradiation was given to 63, chemotherapy to three and irradiation/chemotherapy to four of the patients. After confirmed diagnosis patients were followed until death or September 1, 2009. The median follow-up time for patients still alive was 151 (range; 43 to 378) months. During follow up, 122 (40%) patients died of vulvar cancer. All lesions were staged according to the 2009 International Federation of Gynecology and the Obstetrics (FIGO) classification system [14]. The Regional Committee for Medical Research Ethics South of Norway (S-06012), The Data Inspectorate (04/01043) and The Social and Health Directorate (04/2639 and 06/1478) approved the current study protocol. In this study tumor tissue embedded in paraffin blocks from vulvar cancer patients diagnosed between 1977 and 2006 have been used. As many of these patients are dead or are very old, we did not have the opportunity to obtain patient consent. Permission to perform this study without patient consent, was obtained from The Social and Health Directorate (04/2639).

The histological specimens were reexamined by one of the authors (J.M.N) according to World Health Organization recommendations [15]. Two hundred and eighty (94%) tumors were keratinizing/nonkeratinizing, 13 (5%) were basaloid and 4 (1%) were veruccoid. As controls, samples of normal vulva were collected from 10 patients (age range, 31–65 year) who underwent surgery for benign gynecological diseases. Results from our previous studies on cell cycle proteins using the same cohort of vulvar carcinomas [16-19] were co-analyzed with those of the current study.

### Immunohistochemstry

Three micrometer sections of formalin-fixed, paraffin-embedded tissues were stained immunohistochemically using a Dako EnVision™ Flex + System (K8012; Dako, Glostrup, Denmark) and a Dako Autostainer. Deparaffinization and the unmasking of epitopes were carried out in a PT-Link (Dako) using an EnVision™ Flex target retrieval solution at a high pH (Tris/EDTA pH 9). The tissue sections were incubated with a 0.3% hydrogen peroxide (H_2_O_2_) solution for 5 min to block endogeneous tissue peroxidase activity. Sections were incubated with monoclonal antibody Wee1 (sc-5285, clone B-11, 1:300, 0.67 μg IgG_1_/ml, Santa Cruz Biotechnology Inc., Santa Cruz, CA, USA), and then followed by treatment with EnVision™ Flex + mouse linker (15 min) and EnVision™ Flex/HRP enzyme (30 min). The tissues were stained for 10 minutes with 3′3-diaminobenzidine tetrahydrochloride (DAB), counterstained with hematoxylin, dehydrated and mounted in Richard-Allan Scientific Cyto seal XYL (Thermo Scientific, Waltham, MA, USA). All of the sample series included appropriate positive controls, which included placenta. Negative control included substitution of the monoclonal antibody with mouse myceloma protein of the same subclass and concentration as the monoclonal antibody.

Two observers (R.H. and J.M.N) evaluated the immunostained slides with no knowledge of patient outcome. All discordant scores were reviewed until a final agreement was obtained. Semi-quantitative classes were used to describe the extent of staining (percent of positive tumor cells: absent, 0; < 10%, 1; 10-50%, 2; > 50%, 3) and intensity (absent, 0; weak, 1; moderate, 2; strong, 3). By multiplying the extent and intensity of the signal, product scores for both cytoplasm and nuclear staining were produced ranging from 0 to 9. Protein levels in the nucleus were classified as high when composite scores were ≥6 and low when composite scores were <6. Protein expression in cytoplasm was defined as high when any Wee1 staining was observed and low when no staining was seen. The cutoff value for the immunoreactivity was based on staining pattern observed in normal vulvar epithelium.

### Cell lines and growth conditions

Two human vulvar squamous cell carcinoma cell lines, CAL-39 (DSMZ, Germany) and SW-954 (ATCC, Manassas, VA, USA) were cultured in Dulbecco’s modified Eagle Medium (DMEM, Gibco, LifeTechnologies TM, Invitrogen, Oslo, Norway) supplemented with 10% Fetal Calf Serum (Biocrom, KG, Berlin, Germany) and 2 mM L-glutamine (LONZA, Vervieres, Belgium) and Lonza BioWhittaker L-15 (Leibovitz) Medium (Lonza) containing 20% Fetal Calf Serum and 2 mm L-glutamine, respectively. Both cell lines were grown in monolayer culture at 37°C in humidified conditions containing 5% CO_2_/95% air (CAL-39) or 100% air (SW-954).

### Small interfering RNA (siRNA) transfection

Both cell lines were plated in either 6-well plates (1.5 × 10^5^ cells/well) or in 96-well plates (5 × 10^3^ cells/well) 24 hrs prior to the transfection. The cells were transfected with 25 nM siRNA targeting Wee1 (OligioID; ‘VHS50841’) or RNAi negative control duplexes (Negative Control LOW GC, 12935–200) using Lipofectamine^TM^ RNAiMAX transfection reagents (all reagents from Invitrogen Corporation, CA, USA). Transfection of cells was performed in Opti-MEM® (Invitrogen) for 5 hrs and then replaced with the respective growth medium (described above). Cells were harvested/measured 48 hrs after the transfection was initiated.

### Western blot analysis

Cells were harvested using a rubber policeman, washed once in 1×PBS, and then lysed in ice-cold NP-40 Lysis buffer [(1% NP-40, 10% glycerol, 20 mM Tris–HCl (pH 7.5), 137 mM NaCl, 100 mM NaF), Aprotenin (0.02 mg/mL), Phosphatase inhibitor cocktail 1 (10 μL/mL), Phosphatase inhibitor cocktail 3 (10 μL/mL), Phenyl Methane Sulfonyl Fluoride (PMSF) (1 mM), Leupeptin (0.02 mg/mL), Pepstatin (0.02 mg/mL) and Sodium vanadate (1 mM) (Sigma-Aldrich, St. Louis, MO, USA)], as previously described [10]. Bradford (Bio-Rad Laboratories AB, Sundbyberg, Sweden) analysis was performed for protein quantification, and 25 μg protein/lane was resolved in SDS polyacrylamide gel electrophoresis (PAGE) and transferred to a PDVF immobilon membrane (Millipore, Bedford, MA, USA). To ensure even loading, filters were stained with naphtholblue black (Sigma-Aldrich) and later re-stained with α-tubulin. The membranes were blocked in 5% non-fat milk in TBST (150 mM NaCl, 25 mM Tris-Cl, (pH 7.5), 0.01% Tween 20), and probed with primary antibodies at 4°C overnight, with gentle agitation. Primary antibodies Caspase 3 (#9662/#9664 (even mix)), p21^CIP1/WAF1^ (#2946) and PARP (#9532) were purchased from Cell Signaling (Beverly, MA, USA). α-tubulin (DMIB) was acquired from Calbiochem (Nottingham, UK), whereas Cyclin A (sc-751), p53 (sc-126) and Wee1 (sc-5285) were obtained from Santa Cruz Biotechnology. γ-H2AX (#05-636) was purchased from Millipore, and pCDK1^Tyr15^ (ab47594) and Cyclin B1 (ab32053) antibodies were acquired from Abcam (Cambridge, England). Membranes were thereafter washed 3 × 10 min in TBST. The membranes were subsequently hybridized with an appropriate secondary antibody [HPR-conjugated anti-rabbit or anti-mouse IgG antibodies (Promega)] for 1 hr at room temperature, with gentle agitation, and then washed in TBST for 3 × 10 minutes. Protein bands were visualized after first incubating the membranes with ECL-plus reagent (GE Healthcare, Chalfont St Gils, UK) for 5 min.

### MTS assay

Five thousand cells per well were seeded in 96-well plates and left to attach overnight, before siRNA transfection for the indicated time. Cell viability was determined using the 3-(4,5-dimethylthiazol-2-yl)-5-(3-carboxymethoxyphenyl)-2-(4-sulfophenyl)-2H-tetrazolium) (MTS) assay (Promega, WI, USA), in which the capacity of the cells to convert MTS salt into a brown formazan product was measured. Absorbance was measured at 490 nm using ASYS UVM340 96-well plate reader. Absorbance measured from wells containing medium alone was subtracted, and cell viability was presented as absorbance relative the control.

### Flow cytometric cell cycle analysis

Cells were harvested by trypzination and washed 1 × in PBS. Cell pellets containing approximately 10^6^ cells were re-suspended in 1 mL 70% ice-cold methanol and left to fixate for a minimum of 24 hrs. Fixated cells were washed 1× in PBS, and stained with a solution containing 2 μg/mL Hoechst 33258 in PBS. Flow cytometric analysis was performed using LSR II UV laser (BD biosciences, San Jose, CA), and further processed using FlowJo software (Tree Star, Ashland, OR, USA).

### Statistical analyses

The Pearson’s chi-square (χ^2^) test was used to study the relationship between Wee1 expression and clinicopathologic parameters. Disease-specific survival was calculated from the date of diagnosis to vulvar cancer related death or September 1, 2009, using the method of Kaplan and Meier. The log-rank test was used to compare survival rate. All calculations were processed using SPSS 18.0 statistical software package (SPSS, Chicago, IL, USA) and statistical significance was considered as *P* ≤ 0.05.

## Results

**Figure 1:**
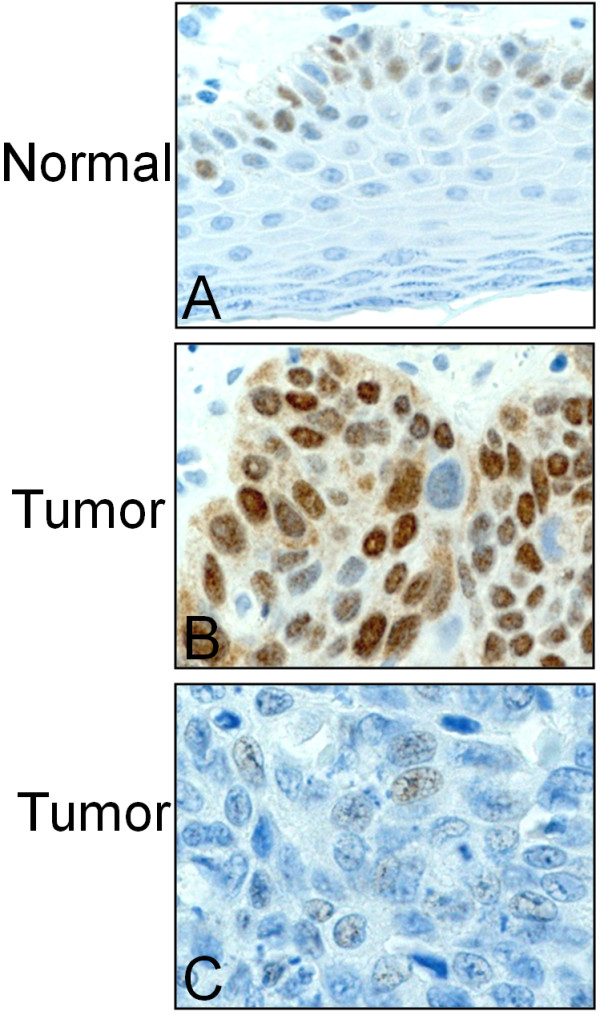
**Expression of Wee1 protein in vulvar squamous epithelium.** Immunohistochemical staining of Wee1 in normal vulvar epithelium **(A)**. High **(B)** and low **(C)** expression of Wee1 in vulvar carcinomas.

In normal vulvar squamous epithelium from 10 patients undergoing surgery for benign gynecological diseases, nuclear staining for Wee1 was identified in basal and parabasal layers (score <6), whereas cytoplasmic staining was not seen (Figure 1A). The immunostaining results in vulvar carcinomas are summarized in Table 1. High Wee1 expression (score ≥6) in the nucleus was identified in 77/297 (26%) of the cases and low levels in 220/297 (74%), whereas, in the cytoplasm positive Wee1 immunoreactivity (score >0) was observed in 157/297 (53%) of the tumors (Figure 1B and C).

**Table 1:** Immunostaining results for Wee1

**Score**	** Nucleus**		** Cytoplasm**	
	**N**	**(%)**	**N**	**(%)**
0	31	(10)	140	(47)
1	5	(2)	18	(6)
2	38	(13)	67	(23)
3	85	(29)	7	(2)
4	61	(20)	45	(15)
6	68	(23)	16	(5)
9	9	(3)	4	(1)
Total	297	(100)	297	(100)

**Figure 2:**
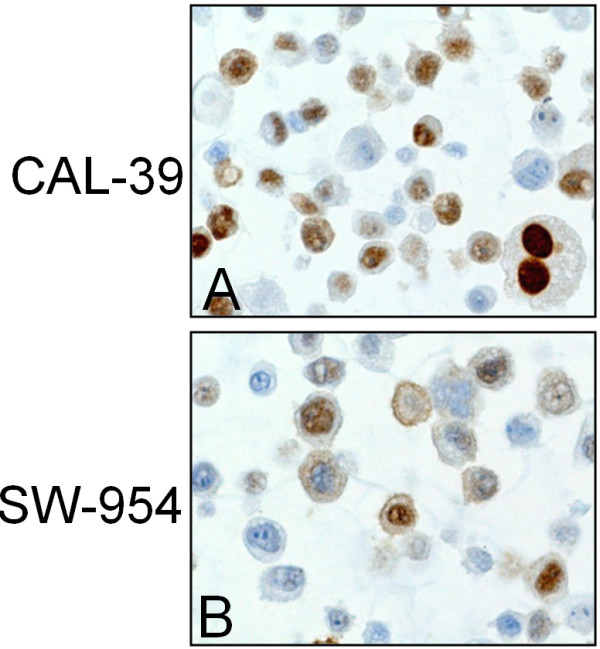
**Expression of Wee1 protein in vulvar cancer cell lines.** Immunohistochemical staining of Wee1 protein in CAL-39 cell line **(A)** and SW-954 cell line **(B)**.

In the vulvar carcinoma cell lines SW-954 and CAL-39 high levels (score ≥6) of nuclear Wee1 immunostaining were observed, additionally, cytoplasmic staining (score =2) was observed in SW-954 cells (Figure 2).

The levels of Wee1 in relation to clinicopathological parameters are shown in Table 2. High expression of Wee1 in the nucleus was significantly correlated with younger age (*P* = 0.01) and presence of lymph node metastasis (*P* = 0.04). Moreover, high expression of Wee1 in the cytoplasm significantly correlated with poor tumor differentiation (*P* = 0.007). High expression of Wee1 in the nucleus significantly correlated with low nuclear and high cytoplasmic level of phospho-CDC25C (Ser216) (*P* = 0.002 and *P* < 0.001, respectively) and high nuclear levels of p21 (*P* = 0.04) and Cyclin A (*P* = 0.004). High Wee1 levels in cytoplasm was significantly correlated with high cytoplasmic levels of CDC25C (*P* = 0.015), 14-3-3β (*P* =0.008), 14-3-3ϵ (*P* = 0.04) and 14-3-3η (*P* = 0.003) (Table 3). By univariate analysis neither nuclear nor cytoplasmic expression of Wee1 were associated with disease-specific survival (*P* = 0.83 and *P* = 0.63).

**Table 2:** Wee1 expression in relation to clinicopathological variables

**Variables**	**Total**	**Nucleus**			**Cytoplasm**		
	**N**	**High**	**(%)**	***P***^**1**^	**High**	**(%)**	***P***^**1**^
Age				0.01			0.27
25-69	117	41	(35)		68	(58)	
70-84	146	27	(18)		74	(51)	
85+	34	9	(26)		15	(44)	
FIGO				0.40			0.12
Ia	10	3	(30)		4	(40)	
Ib	137	27	(20)		73	(53)	
II	13	5	(38)		7	(54)	
IIIa	64	22	(34)		27	(42)	
IIIb	38	11	(29)		23	(60)	
IIIc	12	2	(17)		10	(83)	
IVa	5	1	(20)		1	(20)	
IVb	13	4	(31)		8	(62)	
Not available	5						
Lymph node metastasis				0.04			0.52
None	164	36	(22)		87	(53)	
Unilateral	89	32	(36)		44	(49)	
Bilateral	38	8	(21)		23	(61)	
Not available	6						
Tumor diameter (cm)				0.34			0.09
0.3-2.5	88	17	(19)		39	(44)	
2.6-4.0	93	25	(27)		56	(60)	
4.1-20.0	100	28	(28)		55	(55)	
Not available	16						
Tumor differentiation				0.10			0.007
Well	73	12	(16)		27	(37)	
Moderate	153	45	(29)		87	(57)	
Poor	71	20	(28)		43	(61)	
Depth of invasion (mm)				0.79			0.16
0.0-4.0	76	21	(27)		37	(49)	
4.1-8.0	98	24	(24)		60	(61)	
8.1-40.0	112	26	(23)		56	(50)	
Not available	11						
Infiltration of vessel				0.18			0.67
No	229	55	(24)		120	(52)	
Yes	65	21	(32)		36	(55)	
Not available	3						

^1^Pearson chi-square.

High: Wee1 expression in nucleus ≥ 6 and in cytoplasm > 0.

**Table 3:** Wee1 expression in relation to cell cycle proteins

**Variables**^**1**^	**Total**	**Nucleus**			**Cytoplasm**		
	**N**	**High**	**(%)**	***P***^**2**^	**High**	**(%)**	***P***^**2**^
CDC25C cytoplasm				0.9			0.015
Low (≤ 3)	110	29	(26)		48	(44)	
High (> 3)	187	48	(26)		109	(58)	
Phospho-CDC25C (Ser216) cytoplasm				<0.001			0.18
Low (≤ 3)	147	23	(16)		72	(49)	
High (>3)	150	54	(36)		85	(57)	
Phospho-CDC25C (Ser216) nucleus				0.002			0.16
Low (−)	86	33	(38)		40	(46)	
High (+)	211	44	(21)		117	(55)	
14-3-3β cytoplasm				0.7			0.008
Low (≤ 1)	61	17	(28)		23	(38)	
High (>1)	236	60	(25)		134	(57)	
14-3-3ϵ cytoplasm				1.0			0.04
Low (≤ 1)	42	11	(26)		16	(38)	
High (>1)	255	66	(26)		141	(55)	
14-3-3η cytoplasm				0.07			0.003
Low (≤ 3)	138	29	(21)		60	(43)	
High (>3)	159	48	(30)		97	(61)	
p21 nucleus^3^				0.04			0.5
Low (−)	119	21	(18)		62	(52)	
High (+)	88	26	(30)		50	(57)	
Cyclin A nucleus^3^				0.004			0.2
Low (< 5%)	61	6	(10)		29	(48)	
High (≥ 5%)	146	41	(28)		83	(57)	

^1^Have been studied in previous reports [16-19].

^2^Pearson chi-square.

^3^207 of the 297 vulvar carcinomas have been tested for this marker.

High: Wee1 expression in nucleus ≥ 6 and in cytoplasm > 0.

**Figure 3:**
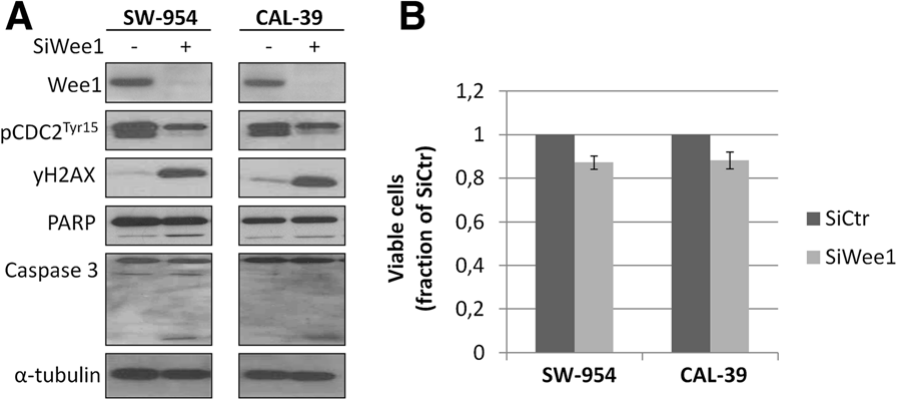
**DNA damages and reduced viability following SiWee1 transfection in vulva cells.** SW-954 and CAL-39 cells were transfected with either SiCtr or SiWee1 (25 nM) and harvested/measured after 48 hrs. **A.** Western blot analysis was conducted with the indicated antibodies. α-tubulin was used as loading control. **B.** Effect of SiWee1 on cell viability as measured by MTS assay. Error bars represent the standard deviation from four independent experiments.

The association between high expression of Wee1 and malignant features in vulvar tumors spurred us to explore how silencing Wee1 would affect the two vulvar cancer cell lines; SW-954 and CAL-39. Wee1 protein expression was effectively removed in both cell lines, along with a reduced expression of the Tyr15 phosphorylation of its downstream target CDK1, as determined by western blotting (Figure 3A). SiRNA mediated silencing of Wee1 led to a marked increase of γ-H2AX, a specific marker of double-strand DNA breaks [20]. Despite the DNA damages, only minute cleavages of the apoptotic markers Caspase 3 and PARP were found in the absence of Wee1. In line with this, transfection with siWee1 only reduced the relative amount of viable cells to approximately 90% of the control cells (Figure 3B).

**Figure 4:**
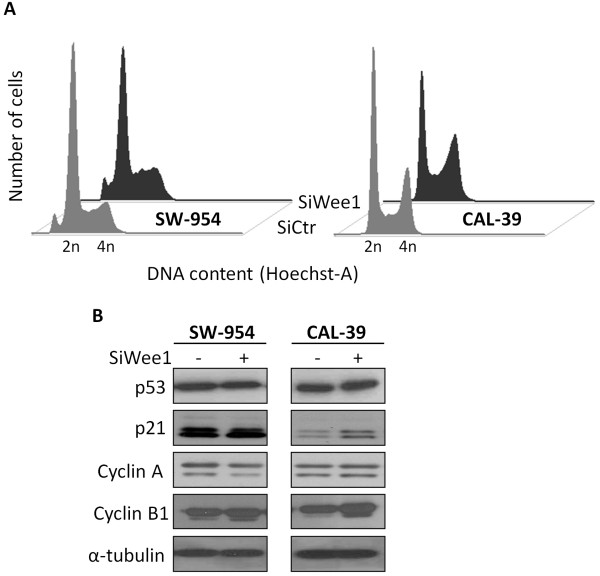
**Effects of SiWee1 on cell cycle distribution and on proteins involved in cell cycle regulation.** SW-954 and CAL-39 cells were transfected with either SiCtr or SiWee1 (25 nM) and harvested after 48 hrs. **A.** Cells were stained with Hoechst and cellular DNA content was measured by Flow cytometry. **B.** Western blot analysis was conducted with the indicated antibodies. α-tubulin was used as loading control. Data are representative of three independent biological experiments.

Given its role in regulating the cell cycle, we next determined the effect of silencing Wee1 on cell cycle distribution and some associated proteins. Only subtle changes in cell cycle distribution were observed following siWee1 transfection, with a minute aggregation of cells in late-S compared to the control cells in both SW-954 and CAL-39 cells (Figure 4A). The latter cell line also displayed an increased amount of p21 protein expression, whereas no changes in p53 levels were seen in either cell line. Furthermore, an augmented expression of Cyclin B1 was found in both cell lines in the absence of Wee1. In SW-954 cells, a weak down-regulation of Cyclin A was observed (Figure 4B).

## Discussion

In the present study we show for the first time that Wee1 is expressed at a higher level in vulvar squamous cell carcinomas compared to normal tissue, and that high expression of the kinase correlates with malignant features including poor histological differentiation and lymph node metastases. In accordance with this, high expression of Wee1 has previously been described in human glioblastoma, osteosarcoma, breast cancer and melanoma [9-12]. Our previous study with melanomas showed a similar association between high Wee1 protein expression and markers of malignancy, as found in vulvar carcinomas [10]. As opposed to our results, a low expression of Wee1 has been described in different studies of breast cancer and melanomas, as well as in non-small-cell lung cancer [13,21,22]. It may be argued that given the roles of Wee1 in stopping the cell cycle in G2/M and in restraining CDK activity during S-phase, low levels of the kinase can possibly facilitate tumor progression. However, the association between high Wee1 expression and the presence of lymph node metastasis as well as poor tumor differentiation found in vulvar cancers does not immediately support the tumor suppressor role of Wee1. Thus, it is possible that Wee1 has a protective function in vulvar carcinomas. By preventing too high CDK activity during S-phase, Wee1 may forestall potentially lethal DNA damages from occurring in the cancer cells. In agreement with this hypothesis, inhibition of Wee1 has led to reduced proliferation in a range of cancer cell lines [10,23-25]. Given the divergent reports on the expression of Wee1 in different cancer forms; the exact role of the kinase in cancer remains largely unknown. The fact that increased expression of Wee1 was associated with lymph node metastasis and poor tumor differentiation indicate that high level of Wee1 may be involved in malignant progression of vulvar carcinomas.

The expression of Wee1 and its association with clinical outcome has only been investigated in a few reports, including one that shows that patients with Wee1 negative non-small-cell lung cancer had a shorter survival than patients with Wee1 positive cancer in univariate-, as well as in multivariate analysis [13]. In our present study, we did not observe any significant association between disease-specific survival and Wee1 expression for patients with vulvar carcinomas. Further studies will be needed to clarify the role of Wee1 as a prognostic marker in human cancer.

Moreover, we found that the association between Wee1 and different cell cycle regulatory proteins depended on their cellular localization. A high expression of nuclear Wee1 correlated with low expression of nuclear- and high level of cytoplasmic phospho-CDC25C (ser216). These findings correspond with the hypothesis that in response to DNA damages, as well as during DNA replication, Chk1 kinase phosphorylates both Wee1 kinase and its complementary counterpart the phosphatase CDC25C [26]. Once phosphorylated, the Wee1 protein stabilizes, thus leading to its subsequent nuclear increase. The Ser216 phosphorylation of CDC25C on the other hand, attracts members of the 14-3-3 family, which facilitates binding to other proteins such as Chk1, Chk2 and c-TAK1, that can bind to and relocate CDC25C to the cytoplasm [27]. Based on this, one could expect the 14-3-3 (β, ϵ, η) proteins to accumulate in the cytoplasm along with phospho-CDC25C (ser216), whilst Wee1 simultaneously would be expressed at a high level in the nucleus. Instead we observed that high cytoplasmic expressions of the 14-3-3 proteins were correlated with high cytoplasmic Wee1, which does not immediately support this notion. However, the 14-3-3 proteins are believed to have several hundred direct binding partners, including many central regulators of the cell cycle, and their cellular localization may thus depend on other factors than Wee1 [27].

Further on, high nuclear expression of Wee1 was associated with high nuclear levels of the S-phase specific Cyclin A protein in vulvar carcinoma samples. Recent studies have demonstrated that Wee1 is required to restrain CDK1 activity during normal S-phase in order to prevent unscheduled initiation of replication forks; hence the kinase expression is thus augmented in this phase of the cell cycle [28]. The association between Wee1 and Cyclin A in vulvar cancer could therefore simply be due to both proteins being expressed in S-phase. Increased Cyclin A has in a previous study been suggested to play a role in the pathogenesis of vulvar squamous cell carcinoma; however no prognostic significance was found [18].

Based on its association with malignancy in vulvar carcinoma samples, we shut down the expression of Wee1 in two vulva squamous cell carcinoma cell lines, SW-954 and CAL-39. The removal of Wee1 protein expression did not affect cell viability to any substantial extent in either cell line. Furthermore, there were no major alterations to cell cycle distribution or cleavage of caspase 3 and PARP, suggesting that the siWee1 treatment neither led to cell cycle arrest nor increased apoptosis. In accordance with these results, inhibition of Wee1 (PD0166285) did not induce cell cycle arrest or cell death when used as mono-treatment in a study with osteosarcoma cell lines [12]. As opposed to this, targeting of Wee1 has in itself been sufficient to cause apoptosis and alterations in cell cycle distribution in other cancer cell lines, including melanoma [10,23,25]. In a study by Iorns et al. where multiple cancer cell lines were screened with an RNAi library (targeting 779 different kinases) in order to identify genes essential for viability, Wee1 was found as a potential target [11]. However, only cell lines displaying a high protein level of Wee1 were responsive to treatment with Wee1 silencing transfections. In the present study, both SW-954 and CAL-39 cell lines showed a high expression of Wee1 when assessed by immunohistochemistry, but regardless of this trait, removal of Wee1 did not translate to any major alteration in viability. Interestingly, despite lack of overall response to siWee1 treatment, a marked increase of γH2AX, indicative of DNA double-strand breaks, was observed in both cell lines. A similar increase in DNA damages following removal of Wee1 activity has been reported in other studies, and may be explained by the proposed role of the kinase in safeguarding the genome during DNA replication [7,8,10,23,25]. Since the vulvar cancer cells did not die or arrest as a result of accumulating DNA damage, it is possible that no crucial genes have been affected or that repair mechanisms are able to correct the damages before the cells undergo mitosis. In support of the latter hypothesis, there appeared to be a very slight increase of cells in late-S phase following knockdown of Wee1. In line with this, an increased expression of Cyclin B, known to accumulate in S-phase and stay high until the end of mitosis, was observed in both cell lines after transfection with Wee1 [29]. The anti-tumor effects of inhibiting Wee1 have been shown as limited to *TP53* mutated cell lines in previous studies, in particular when combined with DNA-damaging agents [30-32]. The proposed rationale for this selected effect is that cells with a dysfunctional G1/S DNA-damage checkpoint, due to *TP53* mutations, are more dependent on stopping in G2 in order to repair DNA damages before entering mitosis. However, cells with functional p53 have also been reported to respond to treatment with inhibitors or siTransfections of Wee1 [10,25]. In a previous study, as many as 44% of vulvar carcinomas were shown to have *TP53* mutations; a large proportion of these also over-expressed p53 protein due to limited degradation as a consequence of structural alterations of the protein [33]. Both cell lines used in this study expressed p53, however no alterations in the protein expression were observed following SiWee1 treatment. CAL-39 did nonetheless show an up-regulation of p21 protein, a downstream target of p53, in the absence of the kinase.

## Conclusion

In conclusion, the association between high Wee1 expression and presence of lymph node metastasis and poor tumor differentiation suggest that Wee1 may be involved in the progression of vulvar carcinomas. However, we found that Wee1 may not function as mono-treatment in these patients.

## Competing interests

Authors declare that they have no competing interests.

## Authors’ contributions

Conceived and designed the experiments: GIM VAF RH. Performed the experiments. GIM EH. Analyzed the data: GIM JMN CGT VAF RH. Contributed reagents/materials/analysis tools: CGT RH. Wrote the paper: GIM VAF RH. All authors read and approved the final manuscript.

## Pre-publication history

The pre-publication history for this paper can be accessed here:

http://www.biomedcentral.com/1471-2407/13/288/prepub
